# Systematical Evaluation of Mechanically Strong 3D Printed Diluted magnesium Doping Wollastonite Scaffolds on Osteogenic Capacity in Rabbit Calvarial Defects

**DOI:** 10.1038/srep34029

**Published:** 2016-09-23

**Authors:** Miao Sun, An Liu, Huifeng Shao, Xianyan Yang, Chiyuan Ma, Shigui Yan, Yanming Liu, Yong He, Zhongru Gou

**Affiliations:** 1 Department of Oral and Maxillofacial Surgery, Second Affiliated Hospital, School of Medicine, Zhejiang University, Hangzhou 310009, Zhejiang, China; 2 Department of Orthopaedic Surgery, Second Affiliated Hospital, School of Medicine, Zhejiang University, Hangzhou, China; 3 Zhejiang Province’s Key Laboratory of 3D Printing Process and Equipment, College of Mechanical Engineering, Zhejiang University, Hangzhou 310027, Zhejiang, China; 4 The State Key Lab of Fluid Power Transmission and Control Systems, College of Mechanical Engineering, Zhejiang University, Hangzhou 310027, Zhejiang, China; 5 Zhejiang-California International Nanosystems Institute, Zhejiang University, Hangzhou 310029, Zhejiang, China

## Abstract

Wollastonite (CaSiO3; CSi) ceramic is a promising bioactive material for bone defect repair due to slightly fast degradation of its porous constructs *in vivo*. In our previous strategy some key features of CSi ceramic have been significantly improved by dilute magnesium doping for regulating mechanical properties and biodegradation. Here we demonstrate that 6 ~ 14% of Ca substituted by Mg in CSi (CSi-Mgx, x = 6, 10, 14) can enhance the mechanical strength (>40 MPa) but not compromise biological performances of the 3D printed porous scaffolds with open porosity of 60‒63%. The *in vitro* cell culture tests *in vitro* indicated that the dilute Mg doping into CSi was beneficial for ALP activity and high expression of osteogenic marker genes of MC3T3-E1 cells in the scaffolds. A good bone tissue regeneration response and elastoplastic response in mechanical strength *in vivo* were determined after implantation in rabbit calvarial defects for 6‒12 weeks. Particularly, the CSi-Mg10 and CSi-Mg14 scaffolds could enhance new bone regeneration with a significant increase of newly formed bone tissue (18 ~ 22%) compared to the pure CSi (~14%) at 12 weeks post-implantation. It is reasonable to consider that, therefore, such CSi-Mgx scaffolds possessing excellent strength and reasonable degradability are promising for bone reconstruction in thin-wall bone defects.

The large demand for bone grafts is increasing with the number of bone defect caused by trauma, inflammation and tumor resection, especially in orthopedic and maxillofacial surgery^1^,^2^. Autologous bone graft is still the gold standard for bone repair, yet it is limited by donor site morbidity, obligatory graft resorption phase and insufficient bone supply^3^. While the heterografts or xenografts obtained from animals also have the problems of poor osteoinductive capability and heterogeneous rejection, which limit their clinical performance^4^,^5^. Ideal artificial biomaterials for bone repair should have good biocompatibility, bioactivity and appropriate degradation rate, which matches bone ingrowth^6^. In addition, the morphology features of the biomaterials also play an important role in the process of bone regeneration, including macropore size, pore interconnectivity and porosity^7^,^8^. Further, the 3D structure of the biomaterials should provide enough mechanical support and restore the custom shape of bone defects^9^.

Artificially synthesized calcium phosphates (CaPs) such as hydroxyapatite (HA), β-tricalcium phosphate (β-TCP) and their biphasic calcium phosphate (BCP), due to their compositional similarity to natural bone mineral, have been applied in clinic. However, poor biodegradability and lack of osteoinductivity are their major drawbacks^10^,^11^,^12^. In the past two decades, a large amount of studies paid attention to Ca-silicate (CSi) ceramics due largely to their exceptional bioactivity and biodegradability^13^. Silicate, which can be combined with ion Ca^2+^, has shown its superiority in pre-osseous and osseous tissue repair *in vitro and vivo*^12^,^13^,^14^,^15^,^16^,^17^. Wollastonite (CaSiO_3_; CSi), which exhibited faster degradation rate and higher osteogenesis, has received a great deal of attention. This ceramics is superior to traditional calcium phosphates in cell attachment, proliferation and differentiation^18^. Some studies have confirmed that bone marrow mesenchymal stem cells and osteoblast-like cells show better proliferation and differentiation on CSi ceramic *in vitro*^19^. In addition, CSi fabricated into porous constructs also presents better bone formation than porous CaPs^12^,^15^. Whereas the pure CSi scaffold undergoes pore-structure collapsing through overfast degradation, and the moisture/water related unfavorably affects its strength stability^12^. During the early stage of bone regeneration, the CSi porous bioceramic could not afford sufficient support to the bone defect. Yet the stability of the early stage is crucial to the bone repair^20^.

Magnesium (Mg), another important biologically essential mineral microelement in bone remodeling, was found to have the ability to enhance bone regeneration of the bone substitute^21^. A variety of Ca-Mg-silicate bioactive glasses and ceramics have been widely investigated for bone regeneration and repair^22^,^23^. We also found that the mechanical properties of CSi ceramic could be significantly enhanced by being doped with limited amount of Mg (Mg substituting Ca by 0–17 mol%: CSi-Mg*x, x* = 0–17). Such CSi-Mg*x* ceramic exhibited significantly improved densification, excellent fracture toughness (>3.2 MPa m^1/2^), and good bioactivity in SBF (simulated body fluid) *in vitro*^24^. Accordingly, it is reasonable to hypothesize that this expected improvement in physicochemical and mechanical properties could qualify 3D porous CSi-Mg*x* bioceramics to reconstruct certain challengeable thin-wall bone defects.

On the other hand, the formation of a fully interconnected macroporous 3D structure is the primary objective in bone scaffold fabrication. Nowadays various techniques have been employed to fabricate isotropic, anisotropic, and periodic pore structure scaffolds, including polymer foam replication^25^, freeze drying^26^, three-dimensional (3D) printing^27^,^28^,^29^ and so on. 3D printing technique shows advantages in designing macropore size, pore interconnectivity and porosity, even the structure of high strength according to the mechanical principles^30^. This technique is also versatile in building up complex constructs with periodic pores and adjustable geometrical parameters for bone repair^31^. The objective of this study is was to systematically evaluate the effect of dilute Mg doping into CSi on osteogenic capacity and mechanical strength of the 3D printed CSi-Mg*x (x* = 6, 10, 14) both *in vitro* and *in vivo.*

## Methods

### Preparation of bioceramic powders

The CSi-Mg*x (x* = 6, 10, 14%) powders with diluted Mg doping were synthesized through a conventional chemical precipitation method previously described^24^, and the pure CSi powders were also prepared in the absence of Mg while the other conditions maintained the same. The powders were ground in a planetary ball miller with 400 RPM to obtain powders with a particle size below 5 μm. The phase composition of ceramic powders was characterized by X-ray diffraction (XRD, Rigaku Co., Japan). Data were collected between 10° and 60° with a step of 0.02° 2*θ* and a dwell time of 1.5 s to identify any crystallization of the powders. The Mg contents in the synthetic powders were measured by inductively coupled plasma atomic emission spectrometer (ICP-AES; Varian Co., USA). Prior to measure, the powders were dissolved in the mixture solutions containing 10% HCl and 10 HNO_3_, respectively.

### Preparation of 3D porous specimens

For layer-by-layer (LbL) ceramic ink writing of the CSi-Mg*x* scaffolds (Ø8 × 3 mm), the ink was prepared by mixing 5.0 g of CSi-Mg*x* powders with 4.5 g of 6% polyvinyl alcohol (PVA) solution. The porous scaffolds were prepared by using 3D ceramic ink writing equipment. The CSi-Mg*x* ink was added to a 5 ml syringe and extruded through a conical nozzle by the movement of a piston rod. A cylindrical porous CSi-Mg*x* scaffold model with 3D rectangular periodic porous architecture was designed using software. The CSi scaffolds were also fabricated simultaneously. The scaffolds using initial distance between green filaments were ~450 μm. The moving speed of the dispensing unit was set to 6 mm/s, and the nozzle diameter was 450 μm. The obtained scaffolds were dried for 24 h in ambient atmosphere, and another 24 h at 90 °C, and then underwent a one-step sintering in a micro-controller controlled temperature furnace (Hefei Kejing Co., China), with temperature increased at the heating rate of 2 °C/min to the target temperature of 1150 °C, held for 3 h and then under natural cooling.

### Physical characterization of CSi-Mg*x* scaffolds

The strut microstructures and the average strut and pore size of the scaffolds were measured through scanning electron microscopy (SEM, JEM-6700F; JEOL). The porosity (macro pores) was measured by Archimedes method in deionized water at room temperature. The ceramic scaffolds were weighed as dry weight (W_1_). Then the scaffolds were immersed in a beaker of water and held under vacuum to make the liquid into the pores until no bubbles emerged from the scaffolds. Subsequently, the samples were re-weighed under water to produce the suspension weight (W_2_). Afterwards, the scaffolds were carefully taken from the beaker with dabbing off surface saturated water, and they were quickly re-weighed in air to produce the saturated wet weight (W_3_). The porosity of the scaffolds (*n* = 5) was calculated via the following equation: porosity (%) = (W_3_ − W_1_)/(W_3_ − W_2_) × 100%.

### Mechanical characterization

Uniaxial compression tests of the ceramic scaffolds were evaluated by a universal testing machine (Instron, 5848 Micro Tester) with a 10 KN load cell and a crosshead-loading rate of 1.0 mm·min^−1^ according to the 5833 ISO standard. Six cylindrical samples (Φ8 mm × 3 mm) were test for each group.

### Cell culture *in vitro*

MC3T3, an osteoblast precursor cell line derived from mouse (Cell Culture Center, Chinese Academy of Medical Science, China) was used to evaluate influence of different materials on proliferation and differentiation. MC3T3 were cultured in Dulbecco’s Modified eagle Medium (DMEM, Gibco, USA) at 37 °C in a humidified atmosphere of 5% CO_2_. Then the cells were detached with 0.25% trypsin, 0.03% ethylene diamine tetraacetic acid (EDTA) and the cell density counted. After counting, the cell suspension was diluted to the desired density in later experiments.

After sterilized by ethylene oxide, four groups of scaffolds were placed into 24-well plate and immersed in DMEM with 10% FBS for 2 h. Then each scaffold was seeded with 1 × 10^5^ MC3T3 cells and incubated at 37 °C in a humidified atmosphere of 5% CO_2_.

### Cell morphology

After 48-hour incubation, samples were washed with phosphate buffer solution (PBS) twice^1^. After fixed with 2.5% glutaraldehyde solution for 2 h, the samples were washed with PBS for three times, treated with 1% osmium tetroxide for 2 h, and then dehydrated in ethanol of ascending concentrations (30, 50, 70, 80, 90, 95, 100 (v/v)) for 5 min respectively. Subsequently, the samples were immersed in isoamyl acetate for 20 min, vacuum-dried at 40 °C for 4 h, and then coated with gold-palladium. Cells morphology on the dried scaffolds was observed using SEM^2^. After fixed with Formalin solution (3.7% formaldehyde in PBS) for 15 min, the fixed samples were washed with PBS twice, and then stained with FITC-Phalloidin (Sigma, St. Louis, USA) for 40 min. Cell nuclei were stained with 2-(4-Amidinophenyl)-6-indolecarbamidine dihydrochloride (DAPI, Beyotime Biotech, Jiangsu, China) solution for 10 min. The samples were observed under a confocal laser scanning microscope (CLSM, Nikon, Japan).

### Cytocompatibility

Cell viability was tested by Cell Counting Kit-8 (CCK-8, Dojindo, Japan) to evaluate the cytocompatibility of the CSi-Mg*x* ceramic scaffolds. Briefly, MG3T3 cells were seeded onto the different CSi-Mg*x* ceramic scaffolds in the 24-well plates and incubated for 1, 4, and 7 d, or cultured in the scaffold-free condition which was served as control, each group containing six samples. All wells were added with 0.5 mL DMEM containing 10% CCK-8 and having incubated for 120 min. The culture media (100 μL) were transferred from the wells to a 96-well plate. The absorbance of the culture media was measured at 570 nm using an MRX Revelation 96-well multiscanner (Dynex Technologies, Chantilly, VA).

### Determination of alkaline phosphatase (ALP) activity

For cell differentiation assay, MG3T3 cells were seeded onto the different CSi-Mg*x* ceramic scaffolds in the 24-well plates and incubated for 14 d, with those cultured in the scaffold-free condition as control. After the culture medium was aspirated, each well was added into with 200 μL of 1% Nonidet P-40 (NP-40) solution and incubated at room temperature for 1 h. The cell lysate was centrifuged and 50 μL of supernatant was transferred to 96-well plates. Fifty milliliters of p-nitrophenylphosphate (Sangon, Shanghai, China) substrate solution (2 mg/mL) composed of 0.1 mol l^−1^ glycine, 1 mmol l^−1^ MgCl. 6H_2_O was added to the 96-well plates and incubated for 30 min at 37 °C. The reaction was quenched by adding 100 mL of 0.1 N NaOH. The absorbance of ALP was quantified at a wavelength of 405 nm using a microplate reader (SPECTRAmax 384, Molecular Devices, USA). The total protein content in the cell lysate was determined using the bicinchoninic acid method in aliquots of the same samples with the Pierce protein assay kit (Pierce Biotechnology Inc., Rockford, IL, USA), read at 562 nm and calculated according to a series of albumin (bovine serum albumin) standards. The ALP levels were normalized to the total protein content, and all the experiments were performed in quadruple.

### Real-time polymerase chain reaction (real-time PCR) analysis

Real-time PCR was used to detect the expression of several osteogenic differentiation-related maker genes (Collagen I (Col I), osteocalcin (OCN), Osterix and runt-related transcription factor 2 (Runx2)) at day 7. Total RNA was extracted using Trizol reagent (Invitrogen) according to the manufacturer’s instructions. The concentration of total RNA was determined using NanoDrop 2000c (Thermo Fisher Scientific Inc. USA). First-stranded complementary DNAs (cDNAs), synthesized from 0.5 μg of the isolated RNA by oligo (deoxythymidine) (oligo (dT)) using DyNamoTM cDNA Sythesis Kit (Fermentas), were applied as templates for real-time PCR. The PCR was performed on a final volume of 25 μL consisting of 1 μL cDNA, 0.5 μL of each primer (forward and reverse), 12.5 μL Power SYBR® Master Mix (2×) (Applied Biosystems, Foster City, CA, USA) and 10.5 μL dd H_2_O with Bio-Rad real-time PCR system (Bio-Rad, Hercules, CA, USA) by using glyceraldehydes-3-phosphatedehydrogenase (GADPH) as the internal control gene for normalization. The forward and reverse primer sequences utilized were listed in Supplementary Table S1. The conditions of real-time PCR were as follows: 95 °C for 1 min followed by 40 cycles at 95 °C for 10 s and 64 °C for 25 s. The comparative expression level was obtained by transforming the logarithmic values into absolute values using 2^−ΔCT = CT(target) − CT(control)^. Then the relative mRNA expression quantification was analyzed with 2^−ΔΔCT^ methods, taking the CSi group as the control.

### Skull defect repair in rabbit model

**Figure 1:**
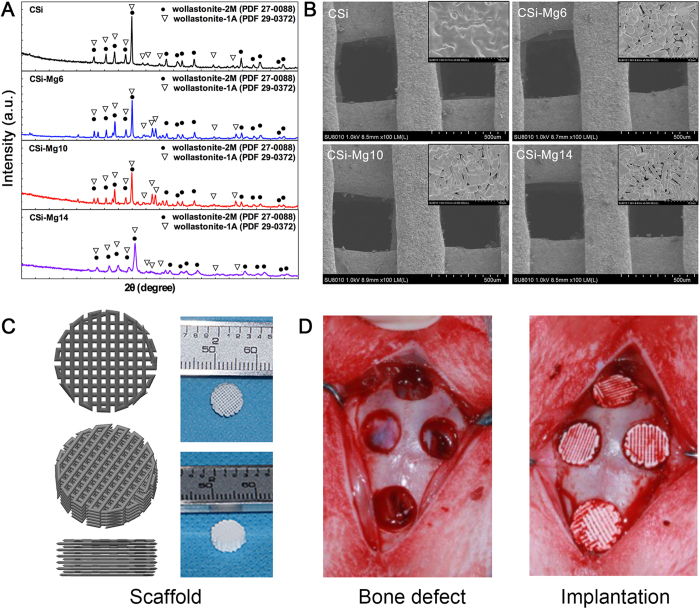
Characterization of CSi-Mg*x* ceramic powders and scaffolds. (**A**) XRD patterns of the ceramic powders. (**B**) The SEM images of surface morphologies and microstructures of the ceramic scaffolds. (**C**) 3D model and macroscopic view of the representative ceramic scaffolds. (**D**) The bone defect and implantation of the ceramic scaffolds in rabbit skull defect.

The *in vivo* study was performed in accordance with the standard animal study guideline of Zhejiang University, and all experimental protocols were approved by the Zhejiang University Ethics Committee (ZJU2014-1-05-093). This study applied 16 New Zealand white rabbits of 2.8 ± 0.2 kg in weight (half males and half females). The procedure was similar to our previous study^2^. Surgery was performed under general anesthesia by intravenously injecting of pentobarbital sodium (30 mg/kg, Sigma). The animal was placed in a prone position and the cranium was shaved and disinfected with povodine iodine. A longitudinal incision was made along the midline from nasal bone to the occipital protuberance and the skin flap was elevated to expose the cranial region. Once the periosteum was removed, four circular bone defects (8 mm in diameter) were created with a dental trephine bur (Fig. 1D). The defects were at least 5 mm away from each other. The four circular defects were randomly filled with four groups of scaffolds and the incision was closed with suture. The rabbits were euthanized with injection of pentobarbital sodium overdose (120 mg/kg, Sigma) 6 or 12 weeks postoperatively. The cranial bones were harvested for further analyses.

### Histological analysis

After the non-invasive MicroCT scanning, the harvested samples (*n* = 3) were dehydrated in graded series of alcohol (80–100%) and embedded in polymethyl methacrylate (PMMA) without decalcification. Three longitudinal sections of approximately 200-μm thickness were cut from each sample using a microtome (SP1600, Leica, Germany). Using a special grinding machine (Exakt-Micro-Grindin System, Leica, Germany), the sections were ground to a thickness of about 40‒50 μm and polished. The sections were stained with Van Gieson’s (V-G) picrofuchsin and examined under light microscopy (DMLA, Leica). The area of newly formed bone was measured by Image Pro 5.0 (Media Cybernetic, USA) and reported as the percentage of the whole bone defect area, respectively. The Newly formed bone (NB) (%) was calculated as follows:


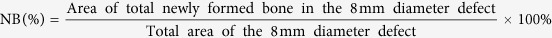


### MicroCT analysis

After the samples (*n* = 6) were cut down from the skull and fixed, MicroCT measurement was performed using a microCT system (vivaCT100, Scanco Medical, Switzerland; 80 kVp, 80 mA) in the parallel to the circular defect. The dataset was processed with Mic View software. The 8 mm-in-diameter circular region of interest (ROI) was traced manually and virtually reconstructed. The area of newly formed bone of ROI was measured. The volume of newly formed bone in the calvarial defects was calculated as BV/TV ratio.

### Mechanical testing

The samples (~8 mm in diameter and ~3 mm in height) were harvested using a trephine drill and dental burr. After the non-invasive MicroCT scanning, the compressive strength of the samples (*n* = 5) was examined using the above-mentioned method (*Materials and Methods 2.4*) at 6 and 12 weeks after implantation, respectively.

### Statistical analysis

All numerical data were expressed as the mean value ± standard deviation (SD). Statistical analysis was performed with one-way analysis of variance (ANOVA), using the SPSS 16.0 for Windows statistics software package. Differences were considered significant at *p* < 0.05.

## Results

### Characterization of CSi-Mgx ceramic powders and scaffolds

The XRD patterns of the CSi-Mg*x* ceramic powders were shown in Fig. 1A. In the spectrum, the CSi, CSi-Mg6, CSi-Mg10 and CSi-Mg14 powders exhibited two types of wollastonite phase including wollastonite-1A (PDF# 29-0732) and wollastonite-2M (PDF# 27-0088), and the latter was increased with the foreign ion doping. SEM images showed the well-defined surface microstructure of the CSi-Mg*x* ceramic scaffolds (Fig. 1B), it displayed the strut diameter (~350 μm) and pore size (~400 μm). The 3D model and macroscopic view of the CSi-Mg*x* ceramic scaffolds were shown in Fig. 1C. With the help of Archimedes method, the porosity of the four groups of bioceramic scaffolds was 60.4 ± 2.7% ~ 63.2 ± 3.1%. In addition, according to ICP measurement, the actual Mg content in the CSi-Mg*x (x* = 6, 10, 14) was 1.28%, 2.09%, and 2.99%, respectively. Thus, these magnesium values in the new synthetic products are close to the theoretical data in the crystalline products of CSi-Mg6 (Ca_0.94_Mg_0.06_SiO_3_; Mg, 1.25%), CSi-Mg10 (Ca_0.90_Mg_0.10_SiO_3_; Mg, 2.12%), and CSi-Mg14 (Ca_0.86_Mg_0.14_SiO_3_; Mg, 2.95%) according to the stoichiometric compounds.

### Compressive strength evaluation

**Figure 2:**
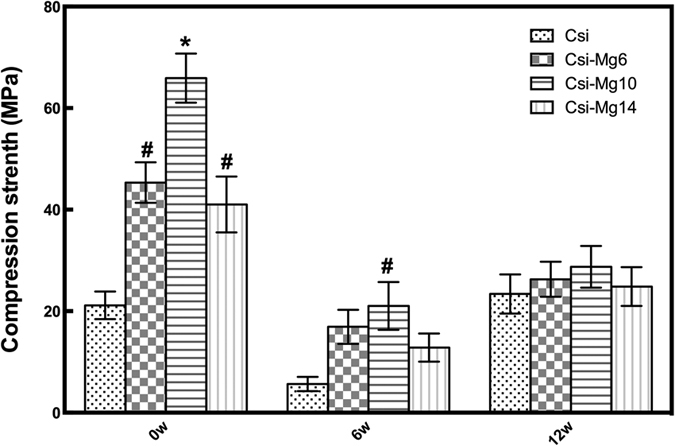
Compressive strength of the CSi and CSi-Mg*x* scaffolds at 0, 6, 12 weeks. *p < 0.05, compared with the other CSi-Mg*x* groups; ^#^*p*  <  0.05, compared with CSi group.

Figure 2 showed the compressive strength of the four groups of original bioceramic scaffolds (week 0) and the samples retrieved 6 weeks and 12 weeks after scaffold implantation. The CSi-Mg10 group showed a highest initial compressive strength (over 65 MPa) among the four groups (0 week; *p* < 0.05). Meanwhile, the CSi-Mg6 and CSi-Mg14 scaffolds also showed a significant higher initial strength (over 40 MPa) than the pure CSi scaffolds (*p* < 0.05). The compressive strength of all groups of scaffolds showed a remarked reduction from 0 to 6 weeks and then an increase at 12 weeks. It is notable that the CSi-Mg10 group still possessed the highest average strength (20.6~27.4 MPa) among the four groups at 6 and 12 weeks and it was significantly higher than that of the CSi group (*p* < 0.05) at 6 weeks.

### Cell culture analysis *in vitro*

#### Cell morphology

**Figure 3:**
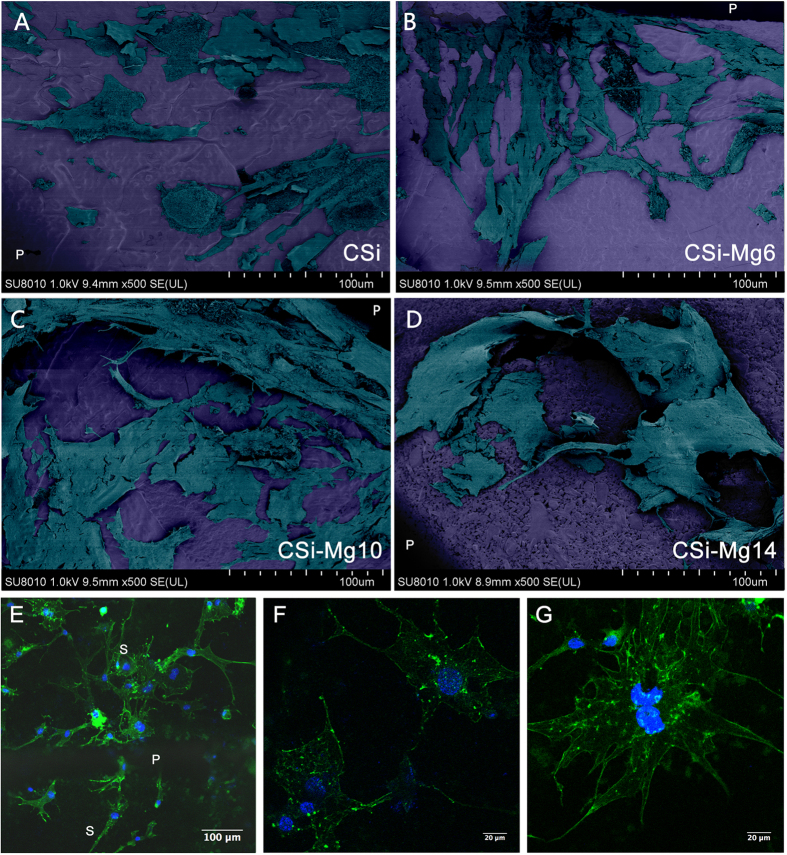
Cell morphology of MC3T3-E1 cells seeded on the bioceramic scaffolds after 48 h under SEM (**A**) CSi; (**B**) CSi-Mg6; (**C**) CSi-Mg10; (**D**) CSi-Mg14) and LSCM microscopy under different magnifications (**E**‒**G**). (**E**) Cytoskeleton stained with FITC-Phalloidin (green) and nuclei stained with DAPI (blue) of cells on the CSi-Mg10 scaffold. P, pore; S, strut. (**F**) MC3T3-E1 cells connecting with each other by filopodia. (**G**) Well-defined microfilaments and cytoskeleton of MC3T3-E1 cells on scaffolds.

Figure 3 showed the SEM and LSCM images of MC3T3 cells seeded on the bioceramic scaffolds after 48 h. As shown in the SEM micrographs, all groups of scaffolds were suitable for MC3T3 cell attachment and the cells had close contact with the scaffolds by filopodia. The cells in the CSi-Mg6, CSi-Mg10 and CSi-Mg14 groups showed more filopodia than that on the pure CSi. The representative LSCM images for the group CSi-Mg10 (Fig. 3E) exhibited that the MC3T3-E1 cells spread well on the scaffolds. The cells attached onto the pore struts and connected to each other closely by filopodia (Fig. 3F). Moreover, the stained cells showed distinct and well-defined microfilaments as well as cytoskeleton (Fig. 3G).

#### Cytocompatibility and osteogenic differentiation *in vitro*

**Figure 4:**
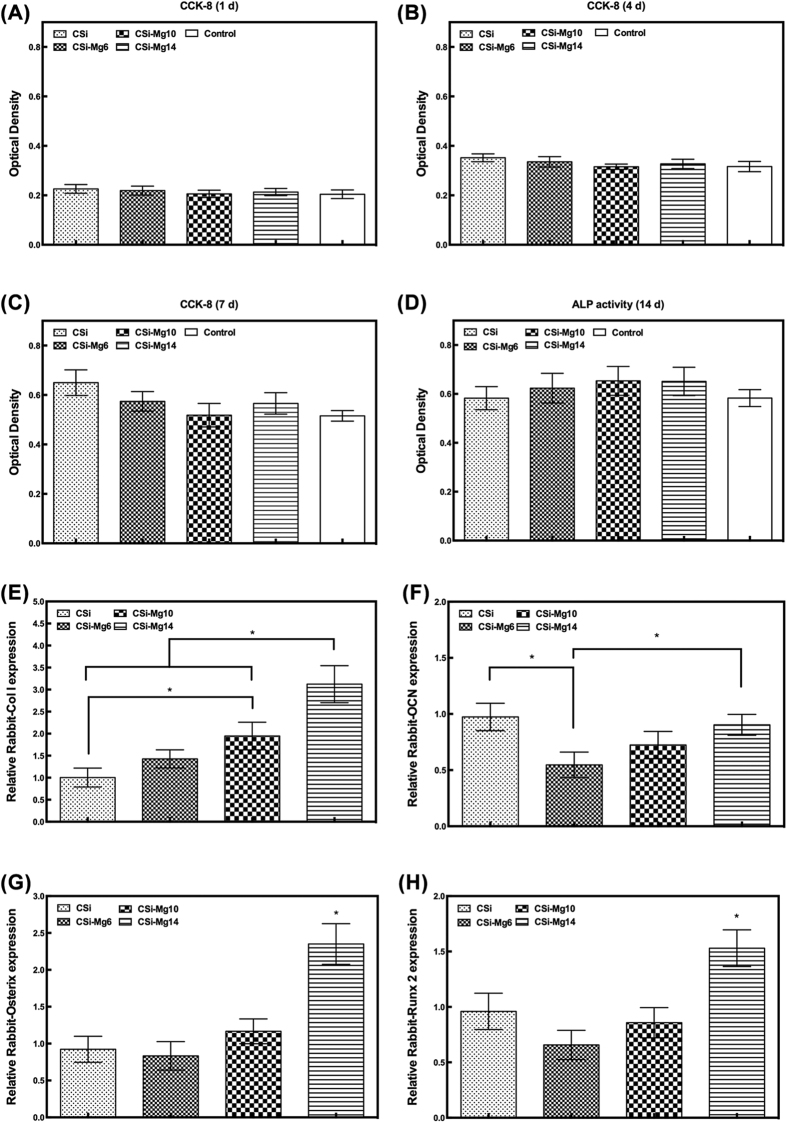
The MC3T3-E1 cell viability after 1 d (**A**), 4 d (**B**), 7 d (**C**) and ALP activity after 7 d (**D**) in CSi-Mg*x* ceramic scaffolds, and the relative expression of osteogenic marker gene COL1 (**E**), OCN (**F**), Osterix (**G**), and Runx2 (**H**) of MC3T3-E1 cells after 7 d (*p < 0.05).

Figure 4A–C showed the viability of MC3T3-E1 cells after incubation on the scaffolds for 1-7 d, with cells cultured directly on the 24-well plate as control. The overall cell viability of the four groups was higher than that of the control, but showed no significant difference among them (*p* > 0.05).

As was shown in Fig. 4D, the CSi-Mg*x* groups showed higher ALP activity than the control. However, no significant difference was found among three groups (*p* > 0.05). Figure 4E–H showed the relative expression of osteogenic genes including Col I, OCN, Osterix, and Runx2 of the osteoblsatic cells after incubation in the CSi-Mg*x* scaffolds for 7 d. The CSi-Mg14 group showed significantly higher relative expression of Col I, Osterix, and Runx2 than the other three groups (*p*  < 0.05) and higher relative expression of OCN than CSi-Mg6 group (*p* < 0.05). The CSi-Mg10 group showed a significantly higher Col I expression than the CSi group (*p* < 0.05). The CSi group showed a significantly higher OCN expression than the CSi-Mg6 group (*p* < 0.05).

### *In vivo* evaluation of skull defect repair

#### Histological analysis

**Figure 5:**
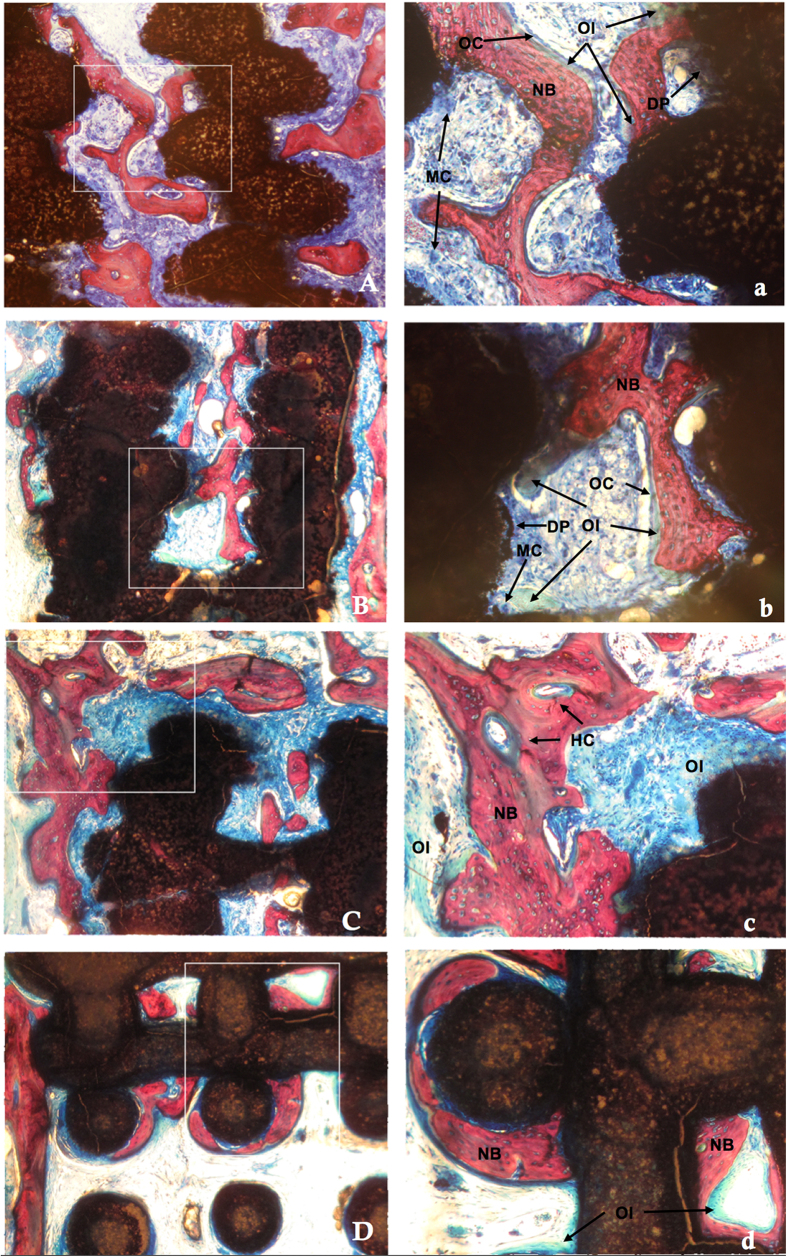
Histological observation of new bone formation in porous scaffolds at 6 weeks (VG staining). (**A**) CSi (**B**) CSi-Mg6 (**C**) CSi-Mg10 (**D**) CSi-Mg14, ×40; (a)CSi (b)CSi-Mg6 (c) CSi-Mg10 (d) CSi-Mg14, ×100. MC, multinucleate cell; BV, blood vessel; DP, degraded particles; OC, osteocytes; OI, osteoid; HC, haversian canal; NB, new bone.

**Figure 6:**
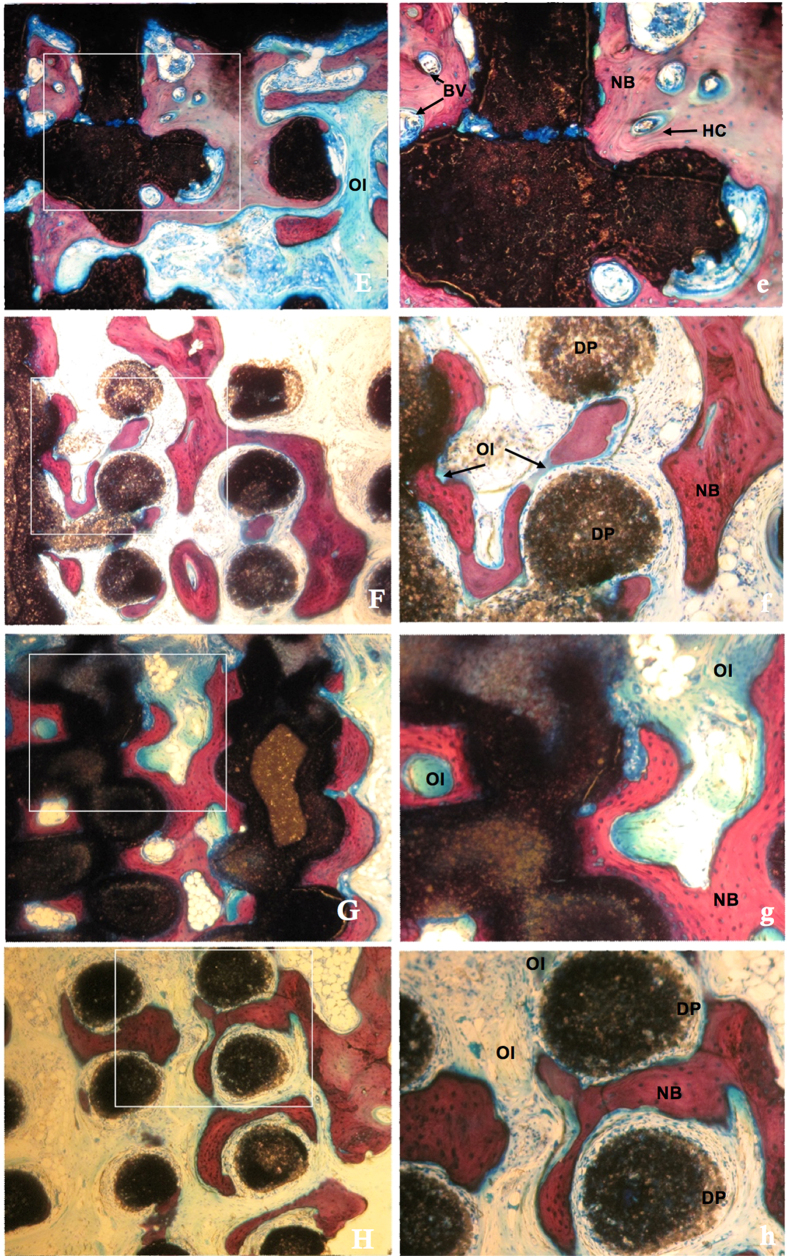
Histological observation of new bone formation in porous scaffolds at 12 weeks (VG staining). (**E**) CSi (**F**) CSi-Mg6 (**G**) CSi-Mg10 (**G**) CSi-Mg14, ×40; (e) CSi (f) CSi-Mg6 (g) CSi-Mg10 (h) CSi-Mg14, ×100. MC, multinucleate cell; BV, blood vessel; DP, degraded particles; OC, osteocytes; OI, osteoid; HC, haversian canal; NB, new bone.

Figures 5 and 6 showed the histological images of the calvarial defects repaired with bioceramics at 6 and 12 weeks post-implantation. The tissue foreign body reaction was weak in all groups, with no inflammatory cells or chronic inflammation processes observed at the bone-biomaterial interface. Biodegradation of the scaffolds was obvious and multinucleate cells were observed around the pore walls in the CSi and CSi-Mg6 scaffolds at week 6. The newly formed bone was observed not only on the surface of the struts but also in the pore of the scaffolds. On the surface of the newly formed bone and biomaterials, more osteoid was observed with osteocytes covering in the CSi-Mg*x (x* = 6, 10, 14) groups than the pure CSi group. In addition, Haversian canals-like structure was also observed. Blood vessels were found in the newly formed bone. More new bone and osteoid formed with time. It is worth mentioning that more bone and osteoid were observed in the skull defects that were repaired with the scaffolds of higher Mg doping.

**Figure 7:**
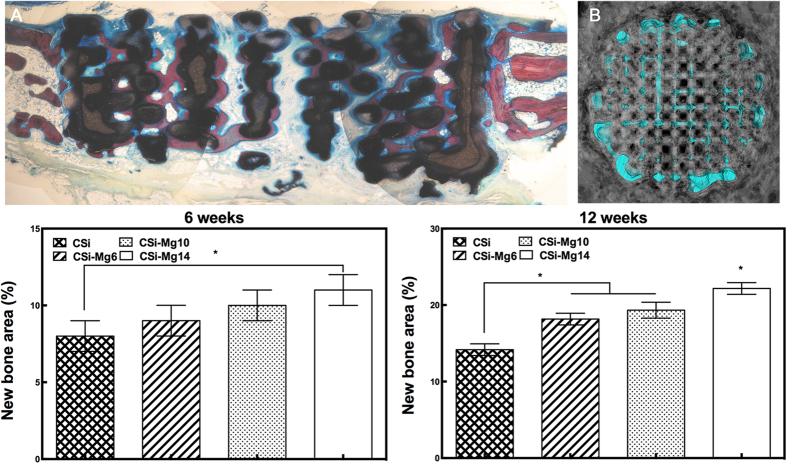
Representative histological observation (**A**) and 3D microCT reconstruction (**B**) of bone defect in CSi-Mg10 scaffold at 12 weeks. Blue, scaffold; gray, bone. (**C**) The percentage of new bone area assessed at weeks 6 and 12 after implantation by histomorphometric analysis. *p < 0.05.

Figure 7 showed the VG stain and 3D reconstruction of the whole bone defect repaired with a CSi-Mg10 scaffold at 12 weeks. The 3D-image was reconstructed with MicroCT software, in which the grey color represented newly formed bone and the blue color represented the 3D print scaffold. Histomorphometric analysis showed that the amount of newly formed bone increased with time (from 6 to 12 weeks) and with the increasing Mg content. At week 6, the percentage of newly formed bone in the CSi-Mg14 group was significantly higher than that in the CSi group (p < 0.05). At week 12, the NB percentage of the CSi-Mgx groups was higher than that of the CSi group (p < 0.05), and that of the CSi-Mg14 group even higher than the other two CSi-Mgx groups (p < 0.05).

#### MicroCT analysis

**Figure 8:**
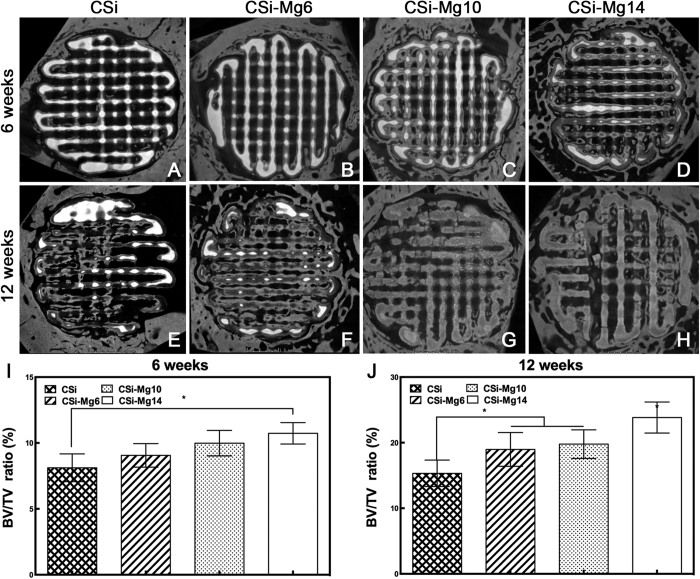
MicroCT images (**A**‒**H**) and quantitative analysis (**I**,**J**) of the volume of the newly formed bone (BV/TV, %) of new bone formation in CSi and CSi-Mg*x* at 6 and 12 weeks, respectively. *p < 0.05.

Figure 8(A–H) showed the MicroCT images of the rabbit circular calvarial defects that were repaired with various bioceramic scaffolds at week 6 and week 12. In all groups, the residual material areas (the highlight areas) decreased over time. Conversely, the new bone areas (the gray areas) increased. As to the contrast between the groups, with more Mg content in the scaffolds, there was less residual material, yet more new bone in the defects. The results (Fig. 8I,J) showed that the CSi-Mg14 group had a significant higher BV/TV ratio than the CSi group (*p* < 0.05) at both week 6 and week 12, higher than the CSi-Mg6 group (*p* > 0.05) and the CSi-Mg10 group (*p* > 0.05) at week 6. The highest BV/TV ratio was found in the CSi-Mg14 group at week 12 (*p* < 0.05). At week 12, the BV/TV ratio in the groups of CSi-Mg6 and CSi-Mg10 were significantly higher than the CSi group (*p* < 0.05). It is worthy of mention that the BV/TV ratio of the CSi-Mg14 group was the highest in both week 6 and week 12. These results were consistent with the histomorphometric analysis.

#### Mechanical testing of retrieved samples

**Figure 9:**
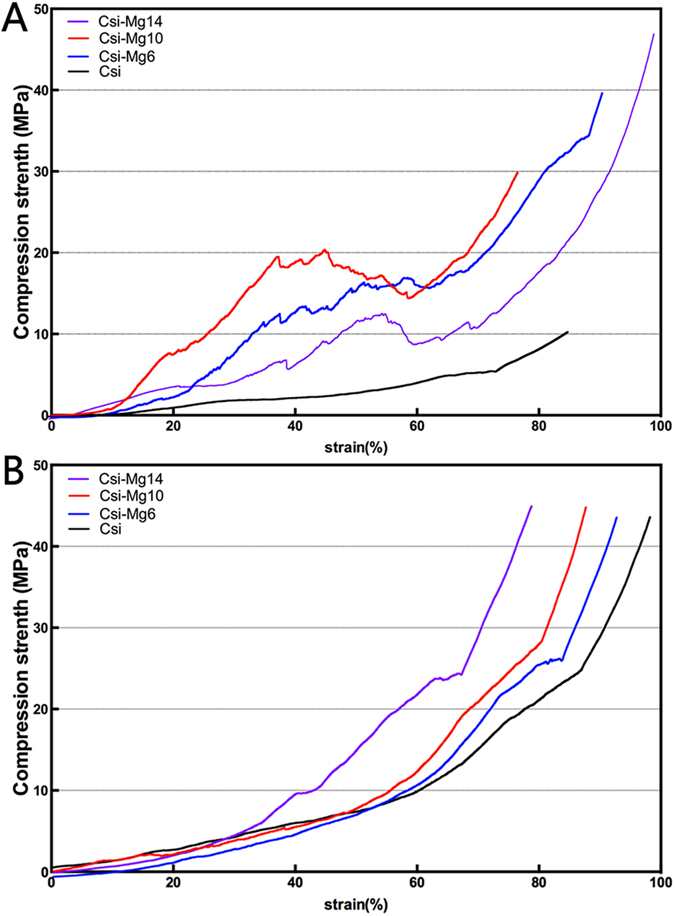
The representative stress-strain curves of the bioceramic scaffolds at 6 weeks (**A**) and 12 weeks (**B**) post-implantation.

Figure 9(A,B) showed the representative compressive strength-deformation curve of the samples retrieved at week 6 and 12. An elastoplastic response was observed in the CSi-Mgx groups at 6 weeks. Initially, the load increased approximately linear with deformation. In the middle stage, a large increase in deformation resulted in a slight change in load. In the final stage, the load increased more steeply, presumably due to compaction of the samples. In contrast, the pure CSi group showed an initially increase with linear deformation in the initial stage and then a linear increase with higher slope in the latter stage, which might also caused by the compaction of the sample. At 12 weeks, all groups showed a nearly three-stage linear curve, in which the slopes of every region increased with the increasing deformation.

## Discussion

Osteoconduction, osseointegration and osteoinduction play an important role in successful bone regeneration of bioactive porous scaffold biomaterial. The biocompatibility and bioactivity of the biomaterials both contribute to this procedure, as well as the morphology of the materials^32^. In this study, all of the osteoblastic cells seeded on the bioceramic scaffolds exhibited good cell viability and ALP activity, as well as the normal cell morphology. The CSi-Mg14 group showed the highest expression of osteogenic marker gene COL1, OCN, Osterix and Runx2 of MC3T3-E1 cells and the highest bone regeneration at week 12 in the rabbit calvarial defect model. As to compression test, the CSi-Mgx groups showed a significant higher compressive strength than the CSi group. The initial CSi-Mg10 scaffolds showed the significant highest compressive strength among all the four groups of scaffolds. In the study of repairing rabbit calvarial bone defect, the samples of the CSi-Mg10 group exhibited significant higher compressive strength than the CSi group at week 6, and remained the highest among all groups at the end of 12 weeks.

The XRD show that the crystallization decreased with addition of magnesium (the XRD line intensity are decreased). Structurally, wollastonite is composed of the repeating unit (Si_3_O_9_)_6_^−^. There are a number of monoclinic and triclinic polytypes of wollastonite, which are as follows: wollastonite-1A, wollastonite-2M, wollastonite-3A, wollastonite-4A, wollastonite-5A, and wollastonite-7A. In compared with the wollastonite-2M (parawollastonite) and wollastonite-7A (pseudwollastonite), wollastonite-1A (wollastonite) is more widely obtained in the polytypes of wollastonite families, and furthermore wollastonite-1A and wollastonite-7A are thought to be the low and high-temperature phase, respectively. In general, the pseudwollastonite belong to high-temperature phase, and the wollastonite-1A and wollastonite-2M are low temperature phase. In particular, the foreign ion doping could helpful for the production of wollastonite-2M phase. In our studies, the magnesium doping readily produced wollastonite-1A, and the amount of wollastonite-2M increase with increasing Mg content.

As mentioned above, the CSi-Mg*x* groups presented the superiority of compressive strength both *in vivo* and *in vitro*. According to our previous studies^24^, the optimized sintering temperature is ~1150 °C at which the CSi-Mgx could produce a high densification but no crystal growth. This is evidently helpful for the mechanical improvement at a conventional pressureless sintering condition for the 3D printing bioceramic constructs. Moreover, the compressive strength of each scaffold group changed with the moles of Mg, consistent with our previous study^24^. The CSi group materials showed the excessive grain growth, which might be the reason for its lower compressive strength (Fig. 1B). Magnesium, which could act as a host grain growth inhibitor^33^ and has higher bond energy with O (Mg-O) than Ca (Ca-O) according to their differences in ion radius, has the capability to increase the compressive strength of the CSi-Mg*x* scaffolds. However, when the mole ratio of Mg content increased to 14%, grain growth is suppressed exceedingly so that the grain needs more energy to bond with each other and form the structure to resist the compression. On the other hand, the 3D printing technology contributes to the orderly pore architectures and high strength of the scaffolds with appropriate macropore size (~400 um)^34^ and appreciate porosity (>60%)^35^. For the reason, scaffolds should be designed in accordance with the mechanical principles to obtain the utmost optimized parameters^30^. The inherent properties of the materials and the manufacturing technique decided together the compressive strength of the four groups of scaffolds in this study.

After implantation *in vivo*, the scaffolds showed a markedly different mechanical response compared with our previous studies *in vitro*^24^,^30^. An elastoplastic response was observed in the CSi-Mgx groups at 6 weeks. In this study, the stress-train curves of the four groups were also different at week 6 and 12 (Fig. 9). This mechanical response of the scaffolds observed in this study is consistent with the recent study in which the porous bioceramics could be altered from a brittle response as fabricated to an elastoplastic response after implantation *in vivo*^36^. It could be inferred that the different mechanical response changed with the different ratio of the newly formed bone and scaffold residuals in the bone defect. From 0 to 12 weeks, the compressive strength of CSi-Mg*x* groups showed an high-low-high change. The compressive strength of the samples of the CSi-Mg*x* groups at week 6 was much lower than that of the initial scaffolds (week 0). Such decrease in compressive strength obviously resulted from material degradation. However, the CSi group only showed two regions at 6 weeks. It might be because the compressive strength of CSi scaffold was similar to the newly formed bone. The compressive strength of the samples of all groups at week 12 was higher than that of samples at week 6. This may be attributed to the new bone in-growth.

All these changes in compressive strength are preferable for the biological procedure of the bone regeneration, especially in segmental bone defect. In general, the bone implants with good compressive strength, for instance, polymethyl methacrylate and titanium, can afford stable support to the defect in the early stage^6^. However, with the time proceeding and the bone growth, the over compressive strength or elastic modulus due to the low degradation or even no degradation leads to significant stress shielding and compromises the integration of the implants in the late stage of bone repair^37^. The CSi-Mg*x* scaffolds performed its high compressive strength (~65 MPa) originally to support the segmental bone defect at early stage^38^. And then, in the following 6 weeks, the compressive strength (~20 Mpa) of the scaffolds decreased but was still higher than the natural cancellous bone (5–10 MPa)^39^. After that, it switches from the scaffold to new bone to provide mechanical support. Eventually, the bioceramics in bone defects would be replaced by the new bone and the mechanical support would be provided by the new bone. The changes in compressive strength of the CSi-Mg*x* scaffolds *in vivo* match the biological procedure of bone regeneration.

As for tissue-engineering, the scaffold, including inherent properties of the materials and its 3D porous constructs, is of specific significance to affect the cells and tissue response. In this study, the Mg-doped Ca-silicate bioceramics ink was easily printed into the expected 3D porous microstructures (Fig. 1C). In the present study, both CSi and CSi-Mg*x* scaffolds showed good cell viability and ALP expression activity, and the CSi-Mg*x* groups showed higher expression of important osteogenic differentiation markers (COL1, OCN, Osterix and Runx2)(Fig. 4). Meanwhile, with the content of Mg increasing from 6 to 14 mol%, the osteogenic gene expression was enhanced. It is reasonable to hypothesize that the ion dissolution products (i.e., Ca, Si, Mg) from the scaffolds have positive effects on bone regeneration. Previous studies have shown that Si and Ca ions improved the osteogenic differentiation of MC3T3 cells^40^,^41^. Moreover, it was confirmed that Mg ions could stimulate the bone marrow stromal cells activity^42^ and promote the fibrocartilage regeneration potential of goat costal chondrocytes^43^. It was also demonstrated that when doped with Mg ions, the 3D porous scaffolds exhibited good cytocompatibility and antibacterial potential *in vitro*^44^. Porous magnesium/PLGA composite scaffolds have been applied in clinic to enhance bone regeneration following tooth extraction *in vivo*^45^. These results are consistent with the bone regeneration capacity of our CSi-Mg*x* scaffolds according to the histological and 3D microCT reconstruction analysis. The newly formed bone has increased with the Mg content in the bioceramic scaffolds at each time point (Figs 7 and 8). Accordingly, we hypothesize that the Mg ions released from the CSi-Mg*x* scaffolds may be one of the key factors in enhancing the osteogenic differentiation *in vitro* and bone regeneration *in vivo*.

In addition, it seems that the osteogenic capacity of CSi-based bioceramic scaffolds is correlated to Mg doping ratio. It has been demonstrated in our previous studies that the higher is the Mg doping ratio, the higher is the densification of the CSi-Mg*x* ceramic and the slower is the dissolution rate *in vitro*^24^. Thus, the improvement of osteogenic capacity for the Mg doped CSi scaffold is possibly achieved by enhancing the densification and inhibiting the biodegradation. In particular, the CSi-Mg10 and CSi-Mg14 scaffolds showed superior bioactivity and mechanical strength to pure CSi scaffolds. Thus, the dilute Mg doping CSi biomaterials have significant potentials for use in thin-wall bone defect repair. Indeed, it remains unknown how the porous scaffolds affect the progress of bone regeneration, and on which further studies are still needed.

## Conclusion

In summary, this research presents for the first time that 3D printed diluted magnesium doping wollastonite porous scaffolds have the superiority of both bone regeneration potential and mechanical evolution in repairing the thin-wall bone defects. The dilute Mg doped CSi scaffolds are superior to the pure CSi scaffolds in viability, ALP activity and expression of osteogenesis-related genes of the osteogenic cells on their surface. The Mg icons in the mechanically strong bioceramics also enhance bone regeneration in the model of rabbit calvarial critical-size bone defect reparation. In this regard, the mechanically strong CSi-Mg scaffolds with appropriate dilute Mg doping is promising for certain scenarios of bone defect reparation, especially the reparation of the thin-wall cranio-maxillofacial bone defects.

## Additional Information

**How to cite this article**: Sun, M. *et al*. Systematical Evaluation of Mechanically Strong 3D Printed Diluted magnesium Doping Wollastonite Scaffolds on Osteogenic Capacity in Rabbit Calvarial Defects. *Sci. Rep.* **6**, 34029; doi: 10.1038/srep34029 (2016).

## Supplementary Material

Supplementary Information
