# Supplementary material for: Systematical Evaluation of Mechanically Strong 3D Printed Diluted magnesium Doping Wollastonite Scaffolds on Osteogenic Capacity in Rabbit Calvarial Defects
Source: Sci Rep. 2016 Sep 23;6:34029. doi: 10.1038/srep34029 (PMC5034319; doi:10.1038/srep34029)
Supplement: Supplementary Information [file srep34029-s1.pdf]

Systematical Evaluation of Mechanically Strong 3D Printed Diluted magnesium Doping Wollastonite Scaffolds on Osteogenic capacity in Rabbit Calvarial Defects

Miao Sun[1], An Liu[2, 4], Huifeng Shao[3,4] , Xianyan Yang[5], Chiyuan Ma[2], Shigui Yan[2], Yanming Liu [1,]*, Yong He[3,4,]*, Zhongru Gou [5,]*

[1]*Department of Oral and Maxillofacial Surgery, Second Affiliated Hospital, School of Medicine, Zhejiang University, Hangzhou 310009, Zhejiang, China.*

[2]*Department of Orthopaedic Surgery, Second Affiliated Hospital, School of Medicine, Zhejiang University, Hangzhou, China.*

[3]*The State Key Lab of Fluid Power Transmission and Control Systems, College of Mechanical Engineering, Zhejiang University, Hangzhou 310027, Zhejiang, China.*

[4] *Zhejiang Province's Key Laboratory of 3D Printing Process and Equipment, College of Mechanical Engineering, Zhejiang University, Hangzhou 310027, Zhejiang, China.*

[5]*Zhejiang-California International Nanosystems Institute, Zhejiang University, Hangzhou 310029, Zhejiang, China.*

Miao Sun, An Liu and Huifeng Shao contributed equally to the work.

*Correspondence and requests for materials should be addressed to Yanming Liu (e-mail: liuyanming@zju.edu.cn), Yong He (e-mail: yongqin@zju.edu.cn)or to Zhong-ru Gou (e-mail: zhrgou@zju.edu.cn).

Supplementary Table S1. The parameters of primers utilized for detecting osteogenetic gene expression.

Gene	Direction	Primer sequence (5`- 3`)
COL1	Forward	GCG GTG GTT ACG ACT TTG GTT
	Reverse	AGT GAG GAG GGT CTC AAT CTG
OCN	Forward	GGC TCA GCC TTC GTG TCC AA
	Reverse	CCC TGC CCG TCG ATC AGT T
Osterix	Forward	GGC ACG AAG AAG CCA TAC TCT GT
	Reverse	GGG AAA AGG CCG GGT AGT CAT
Runx2	Forward	CCC AAG CAT TTC ATC CCT CAC T
	Reverse	CAT ACC GAG GGA CAT GCC TGA
GADPH	Forward	CCG CCT GGA GAA AGC TGC TAA
	Reverse	GAC GAC CTG GTC CTC GGT GTA
